# Associations of egocentric social network characteristics with conversations about the harms of smoking and the benefits of quitting

**DOI:** 10.1371/journal.pone.0344453

**Published:** 2026-07-22

**Authors:** Lizeth Cruz-Jiménez, James F. Thrasher, Victoria C. Lambert, Diego F. Leal, Rachel E. Davis, Chih-Hsiang Yang

**Affiliations:** 1 Department of Health Promotion, Education and Behavior, Arnold School of Public Health, University of South Carolina, Columbia, South Carolina, United States of America; 2 School of Sociology, University of Arizona, Tucson, Arizona, United States of America; 3 Department of Exercise Science, Arnold School of Public Health, University of South Carolina, Columbia, South Carolina, United States of America; Virginia Commonwealth University School of Medicine, UNITED STATES OF AMERICA

## Abstract

**Background:**

Although conversations initiated by smoking-cessation messages are associated with subsequent quit attempts, limited research has examined how the social network characteristics of people who smoke relate to engagement in these conversations. The current study aimed to assess associations between key social network attributes and conversations about the harms of smoking and the benefits of quitting in the context of exposure to novel cessation messages.

**Methods:**

Data were collected from a convenience sample of U.S. adults who smoke (n = 366) and who received a 14-day supply of cigarettes in packs that four different labeling types (i.e., novel text-based messages about smoking harms on the side of packs; pictorial health warnings with the same text, but with imagery and printed on 50% of front and back of packs; efficacy-message inserts with text and imagery about smoking cessation benefits and quitting strategies, placed inside the packs; and/or efficacy message inserts with pictorial health warning labels). At baseline, participants reported their egocentric network characteristics, and each night for 14 consecutive days, they completed a survey about their conversations with network members in the prior 24 hours. Bivariate and adjusted mixed-effects logistic regression models, controlling for experimental message study condition and other covariates, assessed relationships between network attributes and conversations. We estimated separate models predicting the likelihood of conversations (1) about the harms of smoking and (2) about the benefits of quitting on any given day.

**Results:**

In adjusted models, network members’ (i.e., alters’) disapproval of smoking and the prevalence of alters that smoke were positively associated with conversations about both smoking harms and quitting benefits. Network size was negatively associated with, and average closeness to alters was positively associated with, conversations about quitting benefits.

**Conclusion:**

Social network characteristics can influence conversations about smoking harms and cessation benefits. Leveraging these features in cessation interventions could more effectively reduce smoking.

Trial Registration: ClinicalTrials.gov NCT04075682

## Introduction

Social relationships affect mortality risk to a similar degree as other well-established behavioral predictors of mortality, such as cigarette smoking, diet, and physical activity [1,2]. Relationships also influence health behaviors [3], including smoking cigarettes [4,5], with evidence suggesting that the structure and characteristics of social networks influence behaviors and health outcomes through various psychosocial mechanisms [3,6,7].

In research on smoking cessation, one network mechanism that has received some attention is interpersonal communication sparked by cessation messages [8]. These conversations are associated with increased engagement with message content [9] and subsequent quit attempts [9–12] and appear to be one of the pathways through which pictorial health warning labels (PHWLs) discourage smoking [9]. While one previous study used social network methods to examine the characteristics of people with whom people who smoke discuss PHWLs [13], few studies have investigated the social network characteristics associated with conversations about smoking and cessation [8]. To better understand how interventions can increase the occurrence of these conversations, it is critical to identify the social network characteristics that encourage them [14]. In the current paper, we investigate longitudinal associations between selected social network attributes and conversations about the harms of smoking and the benefits of quitting in the context of exposure to novel cessation messages on and/or in cigarette packs.

### Social network characteristics and conversation about smoking and cessation

#### Network size.

People with larger social networks tend to receive more support for personal concerns [15] and be more likely to seek health information from family and friends [16,17]. Thus, people who smoke with larger networks may also be more likely to receive support for smoking cessation and to seek advice for cessation from their networks, which may be operationalized as conversations about smoking and cessation.

#### Tie strength: frequency of communication and perceived closeness.

Tie strength –including frequency of contact with and perceived closeness of network alters (i.e., people in one’s network)– may also influence conversations about smoking and cessation. People who smoke should have more opportunities for these conversations when they talk frequently with their alters. Likewise, closer alters may encourage these conversations, as emotionally closer alters are more likely to provide support for personal matters and health [15]. However, people sometimes avoid conversations about important topics with close alters [18], perhaps out of embarrassment or fear of judgment and rejection from those alters. Because close alters are important, initiating difficult conversations may seem more risky [18], which could lead people who smoke to avoid conversations about their smoking and cessation with stronger alters.

#### Having a significant other.

Trials of PHWLs have found that many people who smoke (34% − 42%) talk to spouses about the warnings [9,19]. Spouses and significant others often live in the same home, communicate frequently, and are often emotionally closer than other network members. Hence, people who smoke with a spouse or significant other will have a person they likely see very frequently and talk to about personal matters, including their health, which may encourage conversations about smoking and cessation. Thus, having a spouse or significant other should be associated with more conversations about smoking and cessation.

#### Smoking disapproval.

Disapproval of smoking within networks of people who smoke also likely affects conversations about smoking and cessation, though it is unclear if this relationship would be negative or positive. The perceptions of others’ approval or disapproval of smoking or cessation have been associated with quitting attempts [20], smoking abstinence [21], quitting intentions [21–23], and other behaviors predictive of quitting [24]. Because conversations about smoking and cessation also predict cessation outcomes, more widespread smoking disapproval among network alters could encourage these conversations.

The only study to investigate the relationship between smoking disapproval and conversations about quitting found that perceived disapproval of smoking was not associated with conversations about quitting [24]. However, that study used a single measure to assess perceived disapproval of smoking and a single measure to assess conversations about quitting with all family or friends [24]. By combining all family and friends, those measures may have imprecisely assessed these constructs, since people may not be accurate at summarizing disapproval or recalling conversations across multiple alters. Further, because the previous study did not assess closeness or frequency of contact with alters, it also may not have captured people who are most likely to influence smoking behavior or who interact with people who smoke regularly enough to have conversations about smoking and cessation. The previous study also asked people to recall conversations from the past week [24], which introduces potential recall bias. Hence, a more nuanced, longitudinal investigation of the relationships between network smoking disapproval and conversations about smoking and cessation is warranted.

Finally, sociological research suggests that smoking disapproval could be negatively associated with conversations about smoking and cessation. People often actively avoid discussing important topics with close alters when they expect the alter will not provide the type of support they are seeking, which may lead to a difficult interaction [18,25]. Hence, to prevent judgment, nagging, or otherwise challenging interactions, people who smoke with close alters who disapprove of their smoking may avoid talking with them about smoking and cessation.

#### Smoking prevalence in networks.

The prevalence of smoking in one’s network may also influence conversations about smoking and cessation. People who smoke and have more network members who smoke tend to experience greater difficulty quitting [26–28]. Therefore, having more people who smoke in one’s network may be negatively associated with conversations about smoking and cessation. However, it is also possible that a higher prevalence of smoking within one’s social network increases conversations about smoking and cessation, as PHWL studies have found that people who smoke discuss PHWLs more often with other people who smoke than with people who do not smoke [9,19]. People often smoke around others who smoke [29], and being around others who are smoking may trigger smoking behavior [30]. As such, smoking sessions with other people who smoke may provide opportunities for social exposure to and discussion of cessation messages on cigarette packs, including more general discussions of smoking and cessation.

Relatedly, having people who formerly smoked in one’s network may encourage conversations about smoking and cessation. Being socially connected to people who formerly smoked appears to promote cessation [5,24,31–33] and some evidence suggests that people discuss important matters with network members they consider to have expertise on the topic being discussed [25]. Moreover, because research suggests that social support and advice from network members is often unsolicited [3,7,34], especially among close alters [35], network members who used to smoke may offer advice on cessation, even when people who smoke do not seek it.

### Study aims and hypotheses

The current study aims to investigate longitudinal associations between the network characteristics described above and conversations about smoking harms and quitting benefits in the context of exposure to novel cessation messages. Our data came from an intensive longitudinal study in which people who smoke in the U.S. reported the conversations in their personal (egocentric) network about smoking and cessation each day over the course of 14 consecutive days. An egocentric network is centered around a single person, or study participant (referred to in network literature as an ‘ego’), while network alters are the people within each person’s network [36]. The current study is among the first to use daily assessments to investigate conversations about smoking and cessation and to use both egocentric social network methods and daily assessments to assess interpersonal communication. Based on the literature described above, we hypothesized that participants’ network characteristics, specifically, larger network size, the presence of people who formerly smoked, and the presence of a spouse or significant other, will be positively associated with the likelihood of having conversations about the harms of smoking and benefits of quitting with network alters. Furthermore, it is expected that participants’ network composition and relational dynamics, specifically, tie strength (i.e., average perceived closeness and frequency of communication), the number of alters who disapprove of smoking, and the number of smokers in the network will be negatively associated with the likelihood of engaging in such conversations.

## Methods

### Sample and recruitment

The present observational study reports secondary analysis of data from a between-subjects, randomized controlled trial conducted between 2019 and 2021 with adults who smoke (n = 366) in South Carolina, North Carolina, and New York, United States. Inclusion criteria were: being ≥18 years old, speaking and reading English, having smoked at least 100 cigarettes in their lifetime, smoking at least 10 cigarettes each day in the prior month, and (during the pre-COVID recruitment period) having an exhaled carbon monoxide level ≥8 ppm to biochemically confirm smoking status. Exclusion criteria included use of other nicotine products in the prior month, due to challenges assessing compensatory behaviors involving other nicotine products when reducing cigarettes.

Multiple recruitment methods were used. In New York State, during the pre-COVID-19 period (i.e., June 28, 2019, to March 5, 2020), a mobile van recruited participants outside smoke shops in low-income neighborhoods across various cities in central New York. In South Carolina, and during the COVID outbreak (i.e., August 13, 2020, to June 29, 2021), participants across all sites were recruited via social media advertisements [37]. Detailed descriptions of the trial design, procedures, and primary outcomes have been reported previously [38]. The parent randomized controlled trial was registered on ClinicalTrials.gov (NCT04075682), and the analysis plan was registered prior to data collection. The study procedures were approved by the University of South Carolina Institutional Review Board (IRB No. Pro00083728) prior to data collection. All participants provided written informed consent electronically via the survey platform prior to participation.

### Study procedures

Participants were randomly assigned to one of four experimental conditions: “Control” (i.e., four novel text-only warning labels on the side of packs); “Insert only” (i.e., inserts, or small, printed leaflets placed inside cigarette packs, with text and imagery on the benefits of smoking cessation and strategies to support quitting, as well as the control text-only warning labels, “PHWL only” (pictorial health warning labels combining graphic images and text describing the harms of smoking, with the same textual content as the text-only warnings); and “PHWL + insert” (both efficacy-message inserts and pictorial health warning labels) (see [38]for further details). Based on self-reported cigarettes per day, participants were given a 14-day supply of their preferred cigarette brand, with packs modified to reflect their experimental condition. All of these were novel relative to current messages on U.S. packs. Full details about the study protocol have been published elsewhere [38].

### Measures

At baseline, participants completed two assessments. They first took a self-administered survey that assessed smoking-related attitudes and behaviors and socio-demographics. They then completed an interviewer-administered survey to assess their social network characteristics. Immediately after the baseline assessments, participants began a 14-day period during which they completed one survey each night to assess conversations about the harms of smoking and the benefits of cessation.

#### Dependent variables (assessed in daily surveys).

*Conversations about the dangers of smoking* and *conversations about the benefits of quitting* with alters were assessed in three steps in the daily surveys. First, the survey asked, “In the last 24 hours, have you talked with [list of alters named at baseline] about good or bad things about smoking or quitting?” (0=No, 1=Yes). Second, if a participant selected “Yes”, the survey repeated the same question for each alter (e.g., “In the last 24 hours, have you talked with [alter 1] about good or bad things about smoking or quitting?” (0=No, 1=Yes)). To reduce potential bias from prompting participants to think about the cigarette labeling messages by constantly asking them about the messages in the nightly surveys, the measures did not explicitly ask about conversations about the cigarette package labels. Third, for each social contact with whom participants discussed smoking or quitting, they indicated the specific conversation topic. Analyses were restricted to discussions of either the harms of smoking or the benefits of quitting, as prior research demonstrates that conversations about quitting smoking and negatively valenced conversations are more strongly linked to quitting outcomes than other discussion types [12,13,39]. Each topic was coded as a dichotomous outcome (0 = not discussed; 1 = discussed).

#### Independent variables (assessed at baseline).

**Network name generator.** To elicit alters, we used a name generator that asked, “Of the people with whom you are closest, who have you spent the most time within the past two weeks?” Participants could name up to 5 people, as some evidence suggests 5 alters is the ideal number to maximize non-redundant network information while minimizing participant burden in network name generators that elicit strong ties [40]. Furthermore, because it is unlikely that young children would have discussions about smoking and cessation, participants were not allowed to name children younger than 3 years old.

The purpose of our network name generator was to elicit alters with whom participants felt close and were likely to interact over the two-week study period. Thus, it was both affect-based and interaction-based [41], which are two popular types of name generators in egocentric network studies [36]. One advantage of having an affect component is that emotionally closer alters are those most likely to influence participants’ attitudes and behaviors [36,42]. Moreover, alters generated by self-reports of interaction are highly reliable over time and are most often long-term alters who are both frequently and recently contacted by participants [43]. Other studies have taken a similar approach of combining affect and interaction name generators into a single name generator [41].

**Network attributes.** To measure alter characteristics, we used an alter-wise ordering approach, assessing all characteristics of each named alter before asking questions about the characteristics of other alters.

*Network size* represents the number of alters each participant named in the name generator, with values ranging from 0–5.

*Tie strength* for each alter was measured with two items. The first item assessed *perceived closeness* and asked participants how close they felt to each alter, with response options ranging from “Not at all close” (1) to “Extremely close” (5). We adapted this item from a Gallup Panel survey that is not publicly available but has been used in published research [44,45]. The second indicator of tie strength was *frequency of communication*, which was assessed by asking participants how often they talked to each alter. Response options included “Every day” (1), “Several times a week” (2), “Once a week” (3), “Once every two weeks” (4), “Once a month or less” (5), and “Don’t know.” We adapted this item from the National Social Life, Health and Aging Project (NSHAP) [46]. “Don’t know” responses were recoded to missing. Then, we reverse coded the responses so that higher values represented more frequent interactions. For each network, we computed averages for perceived closeness and interaction frequency, and we treated these two indicators of tie strength as separate variables in all analyses.

*Having a spouse or significant other* in one’s network was derived from an item adapted from the NSHAP [46] that asked participants the type of relationship they had with each alter. Response options included “Spouse”, “Partner or significant other”, “Parent”, “Child”, “Other family”, “Friend”, “Work colleague”, “Neighbor, “Acquaintance”, and “Other.” Participants could only select one option for each alter. For each network, we created a binary variable to indicate whether each participant named a spouse or significant other (0 = Did not name spouse or significant other; and 1 = Named spouse or significant other).

The smoking status of each alter was assessed by asking, “To the best of your knowledge, does [name of alter] smoke cigarettes?” Response options included “No”, “Not now, but used to smoke”, “Yes”, and “Don’t know.” For each network, we created a variable that counted the number of people who smoke, meaning the number of alters to whom the participant responded “Yes” to the smoking status question (range 0–5 people who smoke). Alters with responses of “No,” “Not now, but used to smoke,” or “Don’t know” were not included. We also created a binary variable to indicate whether each network included a person who formerly smoked, that is, the participant indicated “Not now, but used to smoke” about 1 or more alters (0 = no; 1 = 1 yes).

Smoking approval was assessed for each alter by asking participants, “What does [name of alter] think about your smoking?”. Response options ranged from “Strongly disapproves” (1) to “Strongly approves” (5). We developed this item based on the theories of normative conduct [47] and normative influence [48] as well as measures of smoking disapproval used in prior research [21,24,33]. We dichotomized this variable for each alter to indicate disapproval of smoking, with 1 = Strongly disapproves or Disapproves and 0 = Neutral, Approves, or Strongly approves. We then created a count for each network of the *number of alters who disapproved of smoking* (ranging from 0 to 5).

#### Control variables (assessed at baseline).

*Cigarettes per day* was assessed with an item that asked, “On average, how many cigarettes do you smoke each day, including both factory-made and roll-your-own cigarettes?”

*Recent quit attempt* was measured by asking participants if they had attempted to quit smoking in the past year. Responses were coded as “Yes” or “No.” “Don’t know” responses were classified as missing. Recent quit attempts may predict both future quit attempts and conversations about health warning labels [10,11].

*Intention to quit smoking* was also assessed, and responses were dichotomized to indicate intention to quit within the next six months (1 = Yes; 0 = No), which has been found to predict future quit attempts [49] and conversations about health warning labels [10,11].

*Sociodemographic characteristics and other covariates included: age* (continuous), *sex assigned at birth* (female or male), *race* (White, Black or African American, Latino/Latina/Latinx, Asian, Native Hawaiian or Pacific Islander, American Indian or Alaska Native, or multiple races/ethnicities), *education level* (≤ high school, technical school or some college, or university degree or more) and *income level* (<$30,000; $30,000-$59,000; or ≥$60,000). Because recruitment and data collection methods varied across study sites and pre- and during-COVID-19 periods, all adjusted models controlled for *study site* (New York vs. South and North Carolina) and *study period* (pre- vs. during-COVID-19). *Experimental message condition* (i.e., control, insert only, PHWL only, and PHWL + insert) and *day in study* (day 1- day 14) were also included in all adjusted models.

### Analysis

We used Stata 17 (StataCorp, College Station, TX, USA) for all analyses. For each network variable described above, we created a network-level average or score for each participant. To assess the relationships between network attributes and conversations with alters, we fitted generalized linear mixed models (GLMMs), with a logit link and random intercepts for participants to account for repeated EMA observations nested within participants. Separate models predicted the likelihood of conversations about the harms of smoking and about the benefits of quitting on any given day. For each dependent variable, we estimated bivariate models and two sets of adjusted models. The first set of adjusted models included one primary independent variable (network characteristic) at a time, and all control variables. The second set of adjusted models included all control variables and network characteristics.

For each model, repeated EMA observations (level 1) were nested within participants (level 2), where *i* denotes a given EMA (nightly survey), and *j* denotes a given participant. Generalized linear mixed models (GLMMs) with a logit link and participant-level random intercepts were estimated. The general specification of the model was:

$logit\left(P\left(Y_{ij}=\text{1}\right)\right)=\beta_{\text{0}}+\beta_{\text{1}}X_{\text{1}j}+\ldots +\beta_{p}\;X_{pj}+\gamma_{\text{1}}W_{\text{1}ij}+\ldots +\gamma_{q}W_{qij}+u_{\text{0}j}$

where *Y*_*ij*_ represents whether participant *j* reported a conversation about smoking harms (or cessation benefits, depending on the model) on nightly survey *i*; $\beta_{\text{0}}$ is the fixed intercept; $\beta_{\text{1}}-\beta_{p}\;$ are fixed-effects coefficients for participant-level covariates $X_{\text{1}j}$–$X_{pj}$; $\gamma_{\text{1}}$–$\gamma_{q}$ are fixed-effect coefficients for observation-level covariates $W_{\text{1}ij}$–$W_{qij}$; and 𝑢_0j_ is the participant-specific random intercept that accounts for the clustering of repeated observations within participants. Model diagnostics indicated adequate model convergence and no evidence of problematic multicollinearity among predictors, as assessed using variance inflation factors (VIFs). Inspection of the participant-level random intercepts indicated no substantial departures from normality. Intraclass correlation coefficients estimated separately for each outcome from the fully adjusted models indicated substantial clustering of repeated EMA observations within participants, supporting the inclusion of participant-level random intercepts.

In assessing the extent and potential impact of missingness, we found 14% of the data for the dependent variables were missing, and that network closeness, day in study, education, study period, age, and race were associated with having missing data for the dependent variables. Because missingness was associated with observed covariates, the missing data mechanism was considered consistent with Missing At Random (MAR). Therefore, we used multiple imputation methods [50] to reduce potential bias associated with complete-case analyses and to investigate the effects of missingness on our analyses. We first developed an imputation model that included all primary independent variables from our analysis models, as well as control variables that were associated with missingness of the dependent variables. We then used chained equation methods to produce 20 imputed datasets for each outcome. All hypothesized relationships were re-estimated using imputed datasets, and the resulting estimates were combined using standard multiple-imputation procedures [50]. The direction and significance of associations were consistent across analyses restricted to complete cases and those based on imputed data (S1 Table). Accordingly, we report the results derived from participants with complete information on the key variables, comprising between 348 and 366 individuals.

## Results

### Sample characteristics

Descriptive statistics of study variables are in Table 1. The analytic sample (n = 366) was mostly female (60.4%), White (80.3%), and had greater than a high school education (56.8%). At baseline, most of the sample neither intended to quit smoking in the next six months (66.9%) nor had tried to quit in the prior 12 months (69.4%). The average number of cigarettes smoked per day was 21.6 (SD = 9.20). On average, participants reported talking with alters about the dangers of smoking on one day during the 14-day data collection period (Mean = 1.12, SD = 2.05) and about the benefits of quitting slightly more often (Mean = 1.39, SD = 2.18), with standard deviations exceeding the means, suggesting a right-skewed distribution characterized by many low-frequency responses.

**Table 1:** Key variable descriptive statistics (n= = 366).

	Mean (SD) or %
**Dependent variables**	
Conversations with alters	
About dangers of smoking	1.12 (2.05)
About benefits of quitting	1.39 (2.18)
**Independent variables (network characteristics)**	
Network size (1–5)	3.20 (1.33)
1 alter	13.1%
2 alters	18.3%
3 alters	26.2%
4 alters	19.7%
5 alters	22.7%
Has spouse or significant other	56.6%
Perceived closeness (1 Not at all close – 5 Extremely close)	4.29 (0.62)
Frequency of communication (1 Once a month or less – 5 Every day)	4.64 (0.44)
Number of alters who smoke (0–5)	1.48 (1.13)
Has one or more alters who formerly smoked	23.2%
Number of alters disapproving of smoking (0–5)	1.46 (1.22)
**Control variables**	
Age	44.2 (12.2)
Sex at birth	
Female	60.4%
Male	38.5%
Missing	1.1%
Race	
White	80.3%
Black or African American	12.8%
Latino/Latina/Latinx	2.7%
Asian	0.6%
Native Hawaiian or Pacific Islander	0.3%
American Indian or Alaska Native	1.1%
Multiple races/ethnicities	1.1%
Missing	1.1%
Cigarettes per day	21.6 (9.20)
Recent quit attempt (past year)	
Yes	29.0%
No	69.4%
Missing (includes DK)	1.6%
Intention to quit within 6 months	
Yes	32.2%
No or don’t know	66.9%
Missing	0.8%
Location	
NY	54.9%
SC/NC	45.1%
Study period	
Pre-COVID	55.5%
During COVID	44.5%
Experimental message condition	
Control	27.6%
Inserts only	23.8%
PHWLs only	24.6%
Inserts + PHWLs	24.0%
Education level	
≤ High school	41.8%
Technical school or some college	40.7%
University degree or more	16.1%
Missing	1.4%
Income level	
<$30,000	42.4%
$30,000-$59,000	33.6%
≥$60,000	20.2%
Missing	3.8%

### Correlates of conversations with alters about harms of smoking and benefits of quitting

Table 2 shows the bivariate and adjusted associations between network characteristics and conversations with alters about the harms of smoking. The number of alters disapproving of smoking was positively associated with conversations about the harms of smoking in all three models (OR= 1.29, p < 0.05; AOR = 1.33 p < 0.05; AOR = 1.56, p < 0.01, respectively). The number of alters who smoke was positively associated with these conversations in the model that adjusted for all network and control variables (AOR = 1.49, p < 0.05). Table 3 shows the bivariate and adjusted associations between network characteristics and conversations with alters about the benefits of quitting. Network closeness was positively associated with conversations about the benefits of quitting in all 3 models (OR=1.52, p < 0.05; AOR = 1.65, p < 0.05; AOR = 1.64, p < 0.05 respectively). In the model that adjusted for all network and control variables, network size was negatively associated with conversations about quitting benefits (AOR = 0.71, p < 0.05), and the number of alters who smoke (AOR = 1.62, p < 0.01) and number of alters disapproving of smoking (AOR = 1.43, p < 0.05) were positively associated with these conversations.

**Table 2:** Associations between network characteristics and conversations with alters about the harms of smoking.

	Bivariate models OR (95% CI)^§^		One IV + controls^1^ AOR (95% CI)^§^		Full model (All IVs + controls) ^2^ AOR (95% CI)^§^	
Network size	1.08	(0.89, 1.32)	1.19	(0.96, 1.47)	0.85	(0.60, 1.20)
Has spouse/significant other	1.08	(0.64, 1.80)	1.30	(0.73, 2.32)	1.04	(0.57, 1.91)
Perceived closeness	1.42	(0.92, 2.21)	1.50	(0.93, 2.39)	1.47	(0.86, 2.52)
Frequency of communication	1.15	(0.63, 2.11)	1.09	(0.58, 2.05)	1.10	(0.54, 2.22)
Number of alters who smoke	1.08	(0.86, 1.36)	1.14	(0.90, 1.44)	1.49*	(1.06, 2.08)
Has one or more alters who formerly smoked	1.09	(0.59, 2.03)	1.26	(0.65, 2.43)	1.27	(0.64, 2.54)
Number of alters disapproving of smoking	1.29*	(1.05, 1.60)	1.33*	(1.07, 1.66)	1.56**	(1.12, 2.17)

*p < .05, **p < .01

Notes: Mixed effects logistic regression models were used for all analyses.

OR= odds ratio and AOR = adjusted odds ratio. Adjusted models controlled for day in study, income, education, cigarettes per day, quit attempt in past 12 months, intention to quit within next 6 months, location, pre vs. during COVID, experimental message condition, age, sex at birth, and race.

^1^Each row represents results from a separate adjusted model (network characteristic indicated in the row and all control variables).

^2^Results in this column are from a single model that adjusted for all network characteristics and control variables.

^§^ Sample sizes varied slightly across models because of missing data. For bivariate models, n = 365 for all analyses except “Network size” (n = 366). For adjusted models including one independent variable plus controls, n = 348 for all analyses except “Network size” (n = 349). The fully adjusted model was based on n = 348 participants. All models included participant-level random intercepts. The intraclass correlation coefficient was estimated from the fully adjusted model predicting conversations about harms of smoking and indicated substantial clustering of repeated EMA observations within participants (ICC = 0.51, 95% CI: 0.42–0.59). For the fully adjusted model, AIC = 2186.26 and BIC = 2376.66.

**Table 3:** Associations between network characteristics and conversations with alters about the benefits of quitting.

	Bivariate models OR (95% CI)^§^		One IV + controls^1^ AOR (95% CI)^§^		Full model (All IVs + controls) ^2^ AOR (95% CI)^§^	
Network size	0.98	(0.83, 1.16)	1.01	(0.84, 1.22)	0.71*	(0.53, 0.97)
Has spouse/significant other	1.17	(0.74, 1.86)	1.38	(0.83, 2.28)	1.21	(0.71, 2.06)
Perceived closeness	1.52*	(1.04, 2.23)	1.65*	(1.10, 2.47)	1.64*	(1.03, 2.60)
Frequency of communication	1.06	(0.63, 1.80)	1.09	(0.63, 1.88)	0.90	(0.49, 1.64)
Number of alters who smoke	1.11	(0.90, 1.36)	1.15	(0.94, 1.42)	1.62**	(1.20, 2.19)
Has one or more alters who formerly smoked	1.30	(0.76, 2.23)	1.45	(0.82, 2.55)	1.78	(0.98, 3.23)
Number of alters disapproving of smoking	1.15	(0.95, 1.38)	1.13	(0.94, 1.37)	1.43*	(1.06, 1.91)

*p < .05, **p < .01

Notes: Mixed effects logistic regression models were used for all analyses.

OR= odds ratio and AOR = adjusted odds ratio. Adjusted models controlled for day in study, income, education, cigarettes per day, quit attempt in past 12 months, intention to quit within next 6 months, location, pre vs. during COVID, experimental message condition, age, sex at birth, and race.

^1^Each row represents results from a separate adjusted model (network characteristic indicated in the row and all control variables)

^2^Results in this column are from a single model that adjusted for all network characteristics and control variables.

^§^ Sample sizes varied slightly across models because of missing data. For bivariate models, n = 365 for all analyses except “Network size” (n = 366). For adjusted models including one independent variable plus controls, n = 348 for all analyses except “Network size” (n = 349). The fully adjusted model was based on n = 348 participants. All models included participant-level random intercepts. The intraclass correlation coefficient was estimated from the fully adjusted model predicting conversations about the benefits of quitting and indicated substantial clustering of repeated EMA observations within participants (ICC = 0.44, 95% CI: 0.37–0.52). For the fully adjusted model, AIC = 2592.27 and BIC = 2782.67.

## Discussion

This study investigated how the ego network characteristics of adults who smoke in the United States were related to conversations with alters about smoking harms and cessation benefits, using data from a 14-day daily diary study. We found that people who smoke were more likely to have conversations about the harms of smoking and the benefits of quitting when their social networks included more members who disapproved of smoking and more members who smoked. Additionally, feeling closer to network members was associated with having more conversations about the benefits of quitting. By contrast, larger networks were linked to fewer conversations about the benefits of quitting. Together, these results indicate that both the composition of people who smoke’s personal networks and the quality of their relationships might influence communication about smoking and quitting.

The number of alters disapproving of smoking was positively associated with conversations about both the harms of smoking and the benefits of quitting, which suggests that perceptions of social disapproval are key to encouraging conversations about smoking and cessation that may lead to quitting. Because these conversations can be an important pathway of effect for communication interventions, intervention developers may consider approaches that target social network alters who disapprove of smoking to encourage dialogue about smoking and cessation among people who smoke. More broadly, our findings join a sizable body of research documenting the influence of smoking disapproval on cessation outcomes [20–24,33], which are most effectively changed through policy interventions that have impacts at the population level [20,21,51–54].

The number of network members who smoke was also positively associated with conversations about smoking harms and quitting benefits, which corresponds with prior studies showing that people who smoke discuss PHWLs more often with other people who smoke than with people who do not smoke [19,55]. This contrasts somewhat with other research, which suggests that social connections with people who smoke make it more difficult to quit smoking [26–28]. More research is needed to understand if discussing smoking harms and quitting benefits with other people who smoke actually leads to quitting; however, communication and social network interventions may consider prompting smokers to discuss smoking and cessation among people who smoke.

Average closeness was positively associated with conversations about the benefits of quitting in a bivariate model and adjusted models. However, average closeness was not significantly associated with conversations about the harms of smoking, which suggests close alters are more likely to encourage supportive conversations rather than critical conversations or that people who smoke avoid negative conversations about their smoking with closer alters, which is consistent with research on network mobilization [18]. To better understand whether people who smoke avoid discussing the harms of smoking with close alters, more research is needed that investigates who initiates these conversations. Contrary to our expectations, our other measure of network strength – frequency of communication – was not associated with conversations about smoking harms or quitting benefits. Because the name generator we used elicited names of close alters with whom participants had spoken in the prior 2 weeks, there may not have been sufficient variability in communication frequency to detect a relationship with conversations. Indeed, as shown in Table 1, there was less variability in the frequency of communication with alters compared to our measure of average closeness (see Table 1). While future research should examine these relationships within networks including both weaker and stronger ties, our results suggest perceived closeness is more important than frequency of communication for encouraging conversations that potentially promote cessation.

Our finding that network size was negatively associated with conversations about quitting benefits was contrary to our hypothesis and inconsistent with prior evidence that people who smoke with larger networks report more social support for quitting [15] and are more likely to seek information from friends and family for quitting [16,17]. One reason for these discrepancies is that other studies used either non-truncated network generators (i.e., no limit to network size) or had a much larger limit on network size compared to our study. By restricting the name generator in the current study to five close and frequently contacted alters, we limited respondents’ network size and are unlikely to have captured weaker ties, who may be important discussants in conversations about smoking and cessation. Expansive personal networks that include bridging ties to heterogeneous alters are predicted to facilitate behavioral change; thus, capturing more expansive personal networks in future research is key [56].

Most network characteristics we expected to be associated with conversations about smoking harms and quitting benefits were not associated with having conversations, no matter the topic. This could be because our study was not powered to assess relatively small effect sizes that could characterize associations between network characteristics and conversations. For example, having people who formerly smoked in one’s network was not associated with conversations in our study. By contrast, other studies have found that having social relationships with people who formerly smoked appears to promote cessation outcomes [5,24,32,33], although these studies had much larger samples (i.e., n = 1,571 − 12,067) than our study. Moreover, other studies that have analyzed conversations about smoking and cessation as dependent variables collected data for longer periods of time (e.g., Brewer et al., 2016 assessed conversations for 1 month), so our two-week study had less opportunities to capture these conversations. Future research with larger samples and/or longer study periods may be needed to assess better the relationships between some network attributes and conversations about smoking and cessation.

The findings of this study must be considered in light of some limitations. First, our sample was relatively small and drawn from only three U.S. states, which may have limited statistical power to detect small effects and reduced generalizability; however, the intensive 14-day daily diary design provided time-sensitive data on conversations that minimize recall bias compared to longer recall periods used in prior studies [57]. Second, we did not assess connections among alters, which may have limited our ability to capture broader structural influences on smoking behavior, yet our egocentric approach allowed us to focus on the closest and most influential network members [36]. Third, our convenience sample had higher education and income levels, and a majority of participants were White, but it also included people who smoke from both rural and urban contexts and from multiple regions, thereby increasing diversity compared to many single-site studies. Because network characteristics vary by race, ethnicity, and SES [58–61], future research should examine more representative samples, particularly given the disproportionate burden of smoking-related diseases among people who smoke who are non-White and those with lower SES [62,63]. Finally, although data collection shifted from face-to-face to telephone interviews due to COVID-19, both methods are considered reliable for assessing egocentric networks [64], and while telephone interviews may yield somewhat larger reported network sizes [65], our models adjusted for study period, strengthening confidence in the robustness of findings.

In conclusion, our study provides evidence that disapproval of smoking among social network members of people who smoke and the prevalence of smoking among their contacts influence conversations about smoking and cessation. Other network characteristics may also encourage these conversations, but the evidence for these associations was more limited in our study. Future research should examine how, why, and by whom conversations about smoking and cessation are initiated and whether conversations with specific types of alters are associated with subsequent quitting.

## Supporting information

S1 Table Results using imputed data. (DOCX)
