# Supplementary material for: Associations of egocentric social network characteristics with conversations about the harms of smoking and the benefits of quitting
Source: PLoS One. 2026 Jul 22;21(7):e0344453. doi: 10.1371/journal.pone.0344453 (PMC13390823; doi:10.1371/journal.pone.0344453)
Supplement: S1 Table — (DOCX) [file pone.0344453.s001.docx]

**Supporting information 1. Results using imputed data**

| **Table S1.1 Associations between independent variables and conversations with core ties about the harms of smoking.** | | | | | | | |
| --- | --- | --- | --- | --- | --- | --- | --- |
| **Analysis of imputed data** | | | | | | | |
|  | Bivariate results  OR (95% CI)  n=365-366 | | One IV + controls  AOR (95% CI)^1^  n=348-349 | | Full model (All IVs + controls)  AOR (95% CI) ^2^  n=348 | |  |
| Network size | 1.08 | (0. 91, 1.29) | 1.16 | (0.96, 1.40) | 0.88 | (0.66, 1.18) |  |
| Has spouse/significant other | 1.07 | (0.69, 1.67) | 1.21 | (0.75, 1.97) | 1.00 | (0.61, 1.66) |  |
| Closeness | 1.30 | (0.90, 1.89) | 1.38 | (0.92, 2.05) | 1.38 | (0.89, 2.15) |  |
| Frequency of communication | 1.11 | (0.67, 1.87) | 1.08 | (0.64, 1.84) | 1.14 | (0.64, 2.02) |  |
| Number of network smokers | 1.10 | (0.90, 1.33) | 1.14 | (0.94, 1.39) | **1.43*** | (1.08, 1.90) |  |
| Has one or more former smoker | 1.08 | (0.64, 1.79) | 1.20 | (0.70, 2.05) | 1.22 | (0.70, 2.13) |  |
| Number of alters disapproving of smoking | **1.24*** | (1.04, 1.48) | **1.26*** | (1.04, 1.51) | **1.44*** | (1.09, 1.91) |  |
| *p<.05, **p<.01  Notes: Mixed effects logistic regression models was used to assess relationships between network characteristics and conversations. OR= odds ratio and AOR= adjusted odds ratio. Adjusted models controlled for day in study, income, education, cigarettes per day, quit attempt in past 12 months, intention to quit within next 6 months, location, pre/post COVID, experimental condition, age, sex at birth, and race.  Missing outcome data were addressed using multiple imputation by chained equations. Twenty imputed datasets were generated for each outcome. All hypothesized relationships were re-estimated using the imputed datasets, and the resulting estimates were combined using standard multiple-imputation procedures.  ^1^Each row represents results from a separate adjusted model (network characteristics indicated in the row and all control variables)  ^2^Results in this column are from a single model that adjusted for all network characteristics and control variables. | | | | | | | |

| **Table S1.2 Associations between independent variables and conversations with core ties about the benefits of quitting.** | | | | | | |
| --- | --- | --- | --- | --- | --- | --- |
| **Analysis of imputed data** | | | | | | |
|  | Bivariate results  OR (95% CI)  n=365-366 | | One IV + controls  AOR (95% CI)^1^  n=348-349 | | Full model (All IVs + controls)  AOR (95% CI) ^2^  n=348 | |
| Network size | 0.98 | (0.85, 1.14) | 1.01 | (0.86, 1.18) | **0.75*** | (0.57, 0.98) |
| Has spouse/significant other | 1.15 | (0.77, 1.72) | 1.30 | (0.83, 2.02) | 1.16 | (0.73, 1.84 |
| Closeness | **1.40*** | (1.01, 1.94) | **1.52*** | (1.08, 2.16) | **1.51*** | (1.02, 2.24) |
| Frequency of communication | 1.08 | (0.69, 1.70) | 1.10 | (0.69, 1.75) | 0.97 | (0.58, 1.61) |
| Number of network smokers | 1.10 | (0.93, 1.31) | 1.15 | (0.95, 1.36) | **1.53**** | (1.17, 1.99) |
| Has one or more former smoker | 1.23 | (0.78, 1.94) | 1.34 | (0.82, 2.17) | 1.61 | (0.97, 2.66) |
| Number of alters disapproving of smoking | 1.11 | (0.95, 1.31) | 1.10 | (0.93, 1.30) | **1.35*** | (1.04, 1.75) |
| *p<.05, **p<.01  Notes: Mixed effects logistic regression was used to assess relationships between network characteristics and conversations.  OR= odds ratio and AOR= adjusted odds ratio. Adjusted models controlled for day in study, income, education, cigarettes per day, quit attempt in past 12 months, intention to quit within next 6 months, location, pre/post COVID, experimental condition, age, sex at birth, and race.  Missing outcome data were addressed using multiple imputation by chained equations. Twenty imputed datasets were generated for each outcome. All hypothesized relationships were re-estimated using the imputed datasets, and the resulting estimates were combined using standard multiple-imputation procedures.  ^1^Each row represents results from a separate adjusted model (network characteristics indicated in the row and all control variables)  ^2^Results in this column are from a single model that adjusted for all network characteristics and control variables. | | | | | | |
