# Supplementary material for: Associations of egocentric social network characteristics with conversations about the harms of smoking and the benefits of quitting
Source: PLoS One. 2026 Jul 22;21(7):e0344453. doi: 10.1371/journal.pone.0344453 (PMC13390823; doi:10.1371/journal.pone.0344453)
Supplement: Attachment — Submitted filename: Response to Reviewers.docx [file pone.0344453.s003.docx]

Thank you for taking the time to review our manuscript. We are pleased to present this revised version, which incorporates your valuable feedback. In the following sections, we address each comment and provide clarification where needed. In the updated manuscript, we used the Track Changes tool to make all modifications easy to identify and reference.

**Reviewer #1**

*Throughout*

-Some inconsistencies with capitalization and abbreviations (white vs White, US vs. U.S.)

**RESPONSE:** We reviewed the manuscript and revised inconsistencies in capitalization and abbreviations to ensure adherence to journal style and consistency throughout the text (e.g., “White” and “U.S.”).

-Switch to person first language (people who smoke instead of smokers)

**RESPONSE:** We carefully reviewed the manuscript to use person-first language where appropriate, including replacing terms such as “smokers” with “people who smoke” throughout the document.

*Abstract*

-Can the authors describe what’s in the messages? Are they images or text based? What is the focus of the messages?

**RESPONSE:** We revised and adapted the abstract section to provide additional detail regarding each of the cigarette pack labeling conditions used in the study **(Page 2,** **lines 23-27).**

-Line 29 typo "...network smoking prevalence WAS positively associated..."

**RESPONSE:** We carefully reviewed the sentence and confirmed that the verb agrees with the compound subject (“network disapproval of smoking and network smoking prevalence were positively associated”). The sentence has been retained as written **(Page 2,** **line 36).**

-Did models adjust for study condition?

**RESPONSE:** We thank the reviewer for this important question. Yes, the adjusted models included the experimental message study condition as a control variable. We revised the Methods section in the Abstract to clarify that all adjusted models controlled for study condition and the other covariates included in the analyses (**Page 2,** **lines 30 and 31**).

*Intro*

Line 42- typo ‘influence affect behaviors’ take out ‘affect’

**RESPONSE:** We revised the sentence by removing the word “affect” for clarity and grammatical accuracy (**Page 4,** **line 95**).

Line 142-145- did you hypothesize that the association would be positive or negative?
**RESPONSE:** We thank the reviewer for this helpful suggestion. We revised the hypotheses section to explicitly state the expected direction of associations between network characteristics and conversations about smoking harms and quitting benefits. Specifically, we clarified the hypothesized negative associations for tie strength, smoking disapproval, and network smoking prevalence (**Page 8, line 234**).

*Methods*

-Were alters with ‘don’t know’ responses dropped from number or treated as non-smokers?

**RESPONSE:** We thank the reviewer for this important question. Alters with “Don’t know” responses were not counted as people who smoke in the network smoking variable. Specifically, the variable reflected the number of alters for whom participants responded “Yes” to the smoking status question. We revised the manuscript to clarify this point (**Page 13,** **lines 367-368**).

-Given that the study is from an experiment involving messaging, the authors should provide more detail about the specific messaging used.

**RESPONSE:** We revised the manuscript to provide additional detail regarding the experimental messaging conditions, including the format and focus of the efficacy-message inserts and pictorial health warning labels. Specifically, we clarified that participants in the insert conditions received small printed leaflets placed inside cigarette packs that included both text and imagery focused on the benefits of smoking cessation and strategies to support quitting. We also clarified that the PHWLs combined graphic images with text describing the harms of smoking and contained the same textual content as the text-only warning labels used in the control condition. We also note that the manuscript references the parent trial publication, which provides full details regarding the study protocol and messaging materials (**Page 10,** **lines 275-281**).

-Did authors check for multicollinearity (VIFs) between network size and number of smokers/disapprovers? Should be reported, if so.

**RESPONSE:** We assessed multicollinearity among all predictors, including network size, number of people who smoke within the network, and number of alters who disapprove of smoking, using the variance inflation factor (VIF). The results indicated no evidence of problematic multicollinearity. We have added this information to the Methods section (**Page 15,** **lines 432-434**).

*Results*

-Line 313 typo average NUMBER OF cigarettes
**RESPONSE:** We thank the reviewer for identifying this typo. We revised the text to correct the wording to “average number of cigarettes” (**Page 17,** **line 509).**

*Discussion*

Line 355- missing period at the end of paragraph

**RESPONSE:** We added the missing period at the end of the paragraph **(Page 22, line 656).**

Line 396- typo much LARGER samples

**RESPONSE:** We revised the text to correct the wording to “much larger samples” **(Page 24, line 718).**

**Reviewer #2:**

Line 150-154: The detailed inclusion and exclusion criteria are to be provided.
**RESPONSE:** We thank the reviewer for this suggestion. We revised the manuscript to provide a more detailed description of the inclusion and exclusion criteria. Specifically, we clarified participant eligibility requirements related to age, English language proficiency, smoking history and frequency, and smoking status verification, as well as exclusion criteria related to the use of other nicotine products (**Page 9, lines 244-250**).

Line 261: The coding of this variable is to be clearly stated.

**RESPONSE:** We thank the reviewer for this suggestion. We revised the manuscript to clarify the coding of the recent quit attempt variable. Specifically, responses were categorized as “Yes” or “No,” with “Don’t know” responses classified as missing (**Page 14,** **lines 390-392**).

Line 269: The symbol ; is to be replaced with a comma.

**RESPONSE:** In response to this and the next comment, we revised the text to simplify the presentation of the education categories and replaced semicolons with commas to improve readability (**Page 14,** **line 400**).

Line 270, Table 1: The order of education level is to be revised e.g. ≤ high school; technical school, some college, community college, or university degree or more.
**RESPONSE:** We revised the description of the education categories in the text and **Table 1** to simplify the presentation and align them to improve clarity and consistency (**Page 14, lines 400-401**).

Line 277: Proper citation of the statistical software is to be given e.g. Stata 17 (StataCorp, College Station, TX, USA)
**RESPONSE:** We revised the manuscript to provide the proper citation for the statistical software used in the analyses: “Stata 17 (StataCorp, College Station, TX, USA)” (**Page 15,** **line 413**).

Line 280: The name of the statistical test is to be stated, e.g. the mixed effects logistic regression models were fitted using e.g. GLMM etc

**RESPONSE:** We thank the reviewer for this comment. We clarify that the analyses used generalized linear mixed models (GLMMs) with a logit link (i.e., mixed-effects logistic regression models) and random intercepts for participants to account for repeated EMA observations nested within individuals. We revised the model description and equation to use standard GLMM notation (**Page 15,** **lines 415-417**).

A statement on ‘Model assumptions for the statistical test, e.g., generalized linear mixed models, were satisfied is to be provided.

**RESPONSE:** We thank the reviewer for this suggestion. We revised the manuscript to include a statement regarding the evaluation of model assumptions and diagnostics for the generalized linear mixed models. Specifically, we added information indicating adequate model convergence, no evidence of problematic multicollinearity based on variance inflation factor (VIF) assessments, and no substantial departures from normality in the participant-level random intercepts, as indicated by visual inspection of Q-Q plots. We also clarified that intraclass correlation coefficients estimated separately for each outcome from the fully adjusted models indicated substantial clustering of repeated EMA observations within participants, supporting the inclusion of participant-level random intercepts. These additions have been incorporated into the Methods section (**Pages 15 and 16,** **lines 432-476**).

Line 286-288, 290-294: The paragraph is unclear and requires revision.

**RESPONSE:** The paragraph describing the mixed-effects logistic regression models was revised to improve clarity and consistency with standard generalized linear mixed model (GLMM) notation. Specifically, we clarified the multilevel structure of the data, including the nesting of repeated EMA observations within participants, the specification of participant-level random intercepts, the model equation using conventional GLMM notation, and the inclusion of both participant-level and observation-level covariates (**Page 15, lines 423-432**).

Line 289: The equation is commonly expressed in generalized linear mixed effects model (GLMM). A two-level mixed-effects logistic regression model with a random intercept is specified as logit(P(Yij=1))=β0+β1X1ij+⋯+βkXkij+u0j

**RESPONSE:** We thank the reviewer for this suggestion. We revised the model equation to align with the conventional specification of a generalized linear mixed model (GLMM) for a binary outcome. Specifically, the equation was reformulated using a logit link function and participant-level random intercepts to reflect the mixed-effects logistic regression models estimated in the analyses (**Page 15, line 427**). We also revised the accompanying text to improve clarity and consistency (**Page 15,** **lines 423-432**).

Line 298: The rationale of using multiple imputation is to be stated. Example the type of missingness is to be stated e.g. MAR, MCAR etc

**RESPONSE:** We appreciate this comment and have clarified the rationale for using multiple imputation. Because missingness in the dependent variables was associated with several observed covariates (i.e., network closeness, day in study, education, study period, age, and race), the missing-data mechanism was considered consistent with Missing At Random (MAR). We also clarified that multiple imputation was used to reduce potential bias associated with complete-case analyses and to investigate the effects of missingness on the study findings (**Page 16,** **lines 480-483**). The direction and significance of associations were the same in analyses using only observations with complete data and in analyses using imputed data; therefore, we report the results from the complete-case analyses.

Line 311 and other sections: The decimal point for the percentage figures in the text should be standardized with the figures in the tables.
**RESPONSE:** We revised the manuscript to standardize the presentation of percentage figures in the text, ensuring consistency with the formatting used in the tables. As a consequence, percentages were consistently reported to one decimal place throughout the manuscript (**Pages 16 and 17,** **lines 495-509**) and **Table 1** (**Pages 17 and 18**).

Line 315-316: The SD value is larger than the mean, indicating the probability of skewness.
**RESPONSE:** We acknowledge that the standard deviations exceeded the corresponding means, indicating substantial variability and a likely right-skewed distribution of smoking- and cessation-related conversation frequency. The skewed distribution was acknowledged in the revised Results section (**Page 17,** **lines 513-514**). Because these outcomes reflected low-frequency repeated measures, such variability was expected in this sample.

Table 2 and 3: For the sample size n, indicate clearly which one represents 365, 366, 348, 349.
**RESPONSE:** We thank the reviewer for this suggestion. We revised **Tables 2 and 3** (**Pages 19 and 20**) to clearly indicate the analytic sample sizes associated with each set of models. Specifically, we added table notes that identify the sample sizes for the bivariate, partially adjusted, and fully adjusted models and clarify minor differences across analyses due to missing data.

Random effects, ICC, model fit, etc., if any, are to be reported.
**RESPONSE:** We revised **Tables 2 and 3** (**Pages 19 and 20**) to provide additional information regarding the mixed-effects models. Specifically, we noted that all models included participant-level random intercepts, reported the intraclass correlation coefficients (ICCs) estimated separately for each outcome from the fully adjusted models, and provided model fit statistics (AIC and BIC) for the fully adjusted models. Because Tables 2 and 3 examine different outcomes (conversations about smoking harms and conversations about quitting benefits, respectively), the corresponding ICC values differ across tables. We also clarified this information in the table footnotes.

Line 303, Supplementary Tables S2: Given that multiple imputation was used, clarify how many imputed datasets were generated and whether all datasets were analyzed and pooled? This is to be clearly described.

**RESPONSE:** We thank the reviewer for this suggestion. We revised the manuscript and Supplementary Table S2 (now Supplementary Table S1) to provide additional detail regarding the multiple imputation procedure. Specifically, we clarified that 20 imputed datasets were generated for each outcome using chained equations. We also clarified that all hypothesized relationships were re-estimated using the imputed datasets and that the resulting estimates were combined using standard multiple-imputation procedures (**Page 16, lines 485–488 and Supplementary Tables S1).**

Some references do not conform to the journal’s formatting requirements.

**RESPONSE:** We thank the reviewer for this observation. We carefully reviewed and revised the reference list to improve consistency and ensure conformity with the journal’s formatting requirements.
