# Supplementary material for: Associations of egocentric social network characteristics with conversations about the harms of smoking and the benefits of quitting
Source: PLoS One. 2026 Jul 22;21(7):e0344453. doi: 10.1371/journal.pone.0344453 (PMC13390823; doi:10.1371/journal.pone.0344453)
Supplement: Peer review history

# Peer review: Decision Letter 0

**DOI:** 10.1371/journal.pone.0344453.r001

**Authors:** David Zhu

24 Apr 2026

PONE-D-26-04738

Associations of egocentric social network characteristics with conversations about the harms of smoking and benefits of quitting

PLOS One

Dear Dr. Thrasher,

Thank you for submitting your manuscript to PLOS ONE. After careful consideration, we feel that it has merit but does not fully meet PLOS ONE’s publication criteria as it currently stands. Therefore, we invite you to submit a revised version of the manuscript that addresses the points raised during the review process.

==============================

- I believe this manuscript is interesting and well-written. I believe this will make an important contribution to the literature once methodological considerations highlighted by both reviewers are addressed.

==============================

Please submit your revised manuscript by Jun 08 2026 11:59PM. If you will need more time than this to complete your revisions, please reply to this message or contact the journal office at plosone@plos.org. When you're ready to submit your revision, log on to https://www.editorialmanager.com/pone/ and select the 'Submissions Needing Revision' folder to locate your manuscript file.

Please include the following items when submitting your revised manuscript:

- A letter that responds to each point raised by the academic editor and reviewer(s). You should upload this letter as a separate file labeled 'Response to Reviewers'.
- A marked-up copy of your manuscript that highlights changes made to the original version. You should upload this as a separate file labeled 'Revised Manuscript with Track Changes'
- An unmarked version of your revised paper without tracked changes. You should upload this as a separate file labeled 'Manuscript'.

If you would like to make changes to your financial disclosure, please include your updated statement in your cover letter. Guidelines for resubmitting your figure files are available below the reviewer comments at the end of this letter.

If applicable, we recommend that you deposit your laboratory protocols in protocols.io to enhance the reproducibility of your results. Protocols.io assigns your protocol its own identifier (DOI) so that it can be cited independently in the future. For instructions see: https://journals.plos.org/plosone/s/submission-guidelines#loc-laboratory-protocols. Additionally, PLOS ONE offers an option for publishing peer-reviewed Lab Protocol articles, which describe protocols hosted on protocols.io. Read more information on sharing protocols at https://plos.org/protocols?utm_medium=editorial-email&utm_source=authorletters&utm_campaign=protocols.

As the corresponding author, your ORCID iD is verified in the submission system and will appear in the published article. PLOS supports the use of ORCID, and we encourage all coauthors to register for an ORCID iD and use it as well. Please encourage your coauthors to verify their ORCID iD within the submission system before final acceptance, as unverified ORCID iDs will not appear in the published article. *Only*  the individual author can complete the verification step; PLOS staff *cannot*  verify ORCID iDs on behalf of authors.

We look forward to receiving your revised manuscript.

Kind regards,

David T. Zhu

Academic Editor

PLOS One

Journal Requirements:

When submitting your revision, we need you to address these additional requirements.

1. Please ensure that your manuscript meets PLOS ONE's style requirements, including those for file naming. The PLOS ONE style templates can be found at

https://journals.plos.org/plosone/s/file?id=wjVg/PLOSOne_formatting_sample_main_body.pdf and

https://journals.plos.org/plosone/s/file?id=ba62/PLOSOne_formatting_sample_title_authors_affiliations.pdf.

2. Thank you for stating in your Funding Statement:

“This research was supported by the National Cancer Institute of the National Institutes of Health under Award Number R01 CA215466, awarded to James F. Thrasher. The funders had no role in study design, data collection and analysis, decision to publish, or preparation of the manuscript. “

Please provide an amended statement that declares *all* the funding or sources of support (whether external or internal to your organization) received during this study, as detailed online in our guide for authors at http://journals.plos.org/plosone/s/submit-now.  Please also include the statement “There was no additional external funding received for this study.” in your updated Funding Statement.

Please include your amended Funding Statement within your cover letter. We will change the online submission form on your behalf.

3. We note that you have indicated that there are restrictions to data sharing for this study. For studies involving human research participant data or other sensitive data, we encourage authors to share de-identified or anonymized data. However, when data cannot be publicly shared for ethical reasons, we allow authors to make their data sets available upon request. For information on unacceptable data access restrictions, please see http://journals.plos.org/plosone/s/data-availability#loc-unacceptable-data-access-restrictions.

Before we proceed with your manuscript, please address the following prompts:

a) If there are ethical or legal restrictions on sharing a de-identified data set, please explain them in detail (e.g., data contain potentially identifying or sensitive patient information, data are owned by a third-party organization, etc.) and who has imposed them (e.g., a Research Ethics Committee or Institutional Review Board, etc.). Please also provide contact information for a data access committee, ethics committee, or other institutional body to which data requests may be sent.

b) If there are no restrictions, please upload the minimal anonymized data set necessary to replicate your study findings to a stable, public repository and provide us with the relevant URLs, DOIs, or accession numbers. Please see http://www.bmj.com/content/340/bmj.c181.long for guidelines on how to de-identify and prepare clinical data for publication. For a list of recommended repositories, please see https://journals.plos.org/plosone/s/recommended-repositories. You also have the option of uploading the data as Supporting Information files, but we would recommend depositing data directly to a data repository if possible.

Please update your Data Availability statement in the submission form accordingly.

4. In the online submission form you indicate that your data is not available for proprietary reasons and have provided a contact point for accessing this data. Please note that your current contact point is a co-author on this manuscript. According to our Data Policy, the contact point must not be an author on the manuscript and must be an institutional contact, ideally not an individual. Please revise your data statement to a non-author institutional point of contact, such as a data access or ethics committee, and send this to us via return email. Please also include contact information for the third party organization, and please include the full citation of where the data can be found.

5. We note that Figure S1  includes an image of a [patient / participant / in the study].

As per the PLOS ONE policy (http://journals.plos.org/plosone/s/submission-guidelines#loc-human-subjects-research) on papers that include identifying, or potentially identifying, information, the individual(s) or parent(s)/guardian(s) must be informed of the terms of the PLOS open-access (CC-BY) license and provide specific permission for publication of these details under the terms of this license. Please download the Consent Form for Publication in a PLOS Journal (http://journals.plos.org/plosone/s/file?id=8ce6/plos-consent-form-english.pdf). The signed consent form should not be submitted with the manuscript, but should be securely filed in the individual's case notes. Please amend the methods section and ethics statement of the manuscript to explicitly state that the patient/participant has provided consent for publication: “The individual in this manuscript has given written informed consent (as outlined in PLOS consent form) to publish these case details”.

If you are unable to obtain consent from the subject of the photograph, you will need to remove the figure and any other textual identifying information or case descriptions for this individual.

6. We note that Figure  S1 n your submission contain copyrighted images. All PLOS content is published under the Creative Commons Attribution License (CC BY 4.0), which means that the manuscript, images, and Supporting Information files will be freely available online, and any third party is permitted to access, download, copy, distribute, and use these materials in any way, even commercially, with proper attribution. For more information, see our copyright guidelines: http://journals.plos.org/plosone/s/licenses-and-copyright.

We require you to either (1) present written permission from the copyright holder to publish these figures specifically under the CC BY 4.0 license, or (2) remove the figures from your submission:

a. You may seek permission from the original copyright holder of Figure S1 to publish the content specifically under the CC BY 4.0 license.

We recommend that you contact the original copyright holder with the Content Permission Form (http://journals.plos.org/plosone/s/file?id=7c09/content-permission-form.pdf) and the following text:

“I request permission for the open-access journal PLOS ONE to publish XXX under the Creative Commons Attribution License (CCAL) CC BY 4.0 (http://creativecommons.org/licenses/by/4.0/). Please be aware that this license allows unrestricted use and distribution, even commercially, by third parties. Please reply and provide explicit written permission to publish XXX under a CC BY license and complete the attached form.”

Please upload the completed Content Permission Form or other proof of granted permissions as an "Other" file with your submission.

In the figure caption of the copyrighted figure, please include the following text: “Reprinted from [ref] under a CC BY license, with permission from [name of publisher], original copyright [original copyright year].”

b. If you are unable to obtain permission from the original copyright holder to publish these figures under the CC BY 4.0 license or if the copyright holder’s requirements are incompatible with the CC BY 4.0 license, please either i) remove the figure or ii) supply a replacement figure that complies with the CC BY 4.0 license. Please check copyright information on all replacement figures and update the figure caption with source information. If applicable, please specify in the figure caption text when a figure is similar but not identical to the original image and is therefore for illustrative purposes only.

7. If the reviewer comments include a recommendation to cite specific previously published works, please review and evaluate these publications to determine whether they are relevant and should be cited. There is no requirement to cite these works unless the editor has indicated otherwise.

8. Please review your reference list to ensure that it is complete and correct. If you have cited papers that have been retracted, please include the rationale for doing so in the manuscript text, or remove these references and replace them with relevant current references. Any changes to the reference list should be mentioned in the rebuttal letter that accompanies your revised manuscript. If you need to cite a retracted article, indicate the article’s retracted status in the References list and also include a citation and full reference for the retraction notice.

Additional Editor Comments (if provided):

I believe this manuscript is interesting and well-written. I believe this will make an important contribution to the literature once methodological considerations highlighted by both reviewers are addressed.

[Note: HTML markup is below. Please do not edit.]

Reviewers' comments:

Reviewer's Responses to Questions

**Comments to the Author**

1. Is the manuscript technically sound, and do the data support the conclusions?

The manuscript must describe a technically sound piece of scientific research with data that supports the conclusions. Experiments must have been conducted rigorously, with appropriate controls, replication, and sample sizes. The conclusions must be drawn appropriately based on the data presented.

Reviewer #1: Yes

Reviewer #2: Partly

**********

2. Has the statistical analysis been performed appropriately and rigorously?

Reviewer #1: Yes

Reviewer #2: No

**********

3. Have the authors made all data underlying the findings in their manuscript fully available?

The PLOS Data policy requires authors to make all data underlying the findings described in their manuscript fully available without restriction, with rare exception (please refer to the Data Availability Statement in the manuscript PDF file). The data should be provided as part of the manuscript or its supporting information, or deposited to a public repository. For example, in addition to summary statistics, the data points behind means, medians and variance measures should be available. If there are restrictions on publicly sharing data—e.g. participant privacy or use of data from a third party—those must be specified.

Reviewer #1: No

Reviewer #2: Yes

**********

4. Is the manuscript presented in an intelligible fashion and written in standard English?

PLOS ONE does not copyedit accepted manuscripts, so the language in submitted articles must be clear, correct, and unambiguous. Any typographical or grammatical errors should be corrected at revision, so please note any specific errors here.

Reviewer #1: Yes

Reviewer #2: Yes

**********

5. Review Comments to the Author

Please use the space provided to explain your answers to the questions above. You may also include additional comments for the author, including concerns about dual publication, research ethics, or publication ethics. (Please upload your review as an attachment if it exceeds 20,000 characters)

Reviewer #1: Thanks for the opportunity to review this manuscript! It is very well-written and timely, providing important information about tailoring interventions for smoking cessation to account for social network dynamics. I have a few comments to strengthen the manuscript:

Throughout

-Some inconsistencies with capitalization and abbreviations (white vs White, US vs. U.S.)

-switch to person first language (people who smoke instead of smokers)

Abstract

-Can the authors describe what’s in the messages? Are they images or text based? What is the focus of the messages?

-Line 29 typo "...network smoking prevalence WAS positively associated..."

-Did models adjust for study condition?

Intro

Line 42- typo ‘influence affect behaviors’ take out ‘affect’

Line 142-145- did you hypothesize that the association would be positive or negative?

Methods

-Were alters with ‘don’t know’ responses dropped from number or treated as non-smokers?

-Given that the study is from an experiment involving messaging, the authors should provide more detail about the specific messaging used.

-Did authors check for multicollinearity (VIFs) between network size and number of smokers/disapprovers? Should be reported, if so.

Results

-Line 313 typo average NUMBER OF cigarettes

Discussion

Line 355- missing period at the end of paragraph

Line 396- typo much LARGER samples

Reviewer #2: The manuscript requires improvement.

Line 150-154: The detailed inclusion and exclusion criteria are to be provided.

Line 261: The coding of this variable is to be clearly stated.

Line 269: The symbol ; is to be replaced with a comma.

Line 270, Table 1: The order of education level is to be revised e.g. ≤ high school; technical school, some college, community college, or university degree or more.

Line 277: Proper citation of the statistical software is to be given e.g. Stata 17 (StataCorp, College Station, TX, USA)

Line 280: The name of the statistical test is to be stated, e.g. the mixed effects logistic regression models were fitted using e.g. GLMM etc

A statement on ‘Model assumptions for the statistical test, e.g., generalized linear mixed models, were satisfied is to be provided.

Line 286-288, 290-294: The paragraph is unclear and requires revision.

Line 289: The equation is commonly expressed in generalized linear mixed effects model (GLMM). A two-level mixed-effects logistic regression model with a random intercept is specified as logit(P(Yij=1))=β0+β1X1ij+⋯+βkXkij+u0j

Line 298: The rationale of using multiple imputation is to be stated. Example the type of missingness is to be stated e.g. MAR, MCAR etc

Line 311 and other sections: The decimal point for the percentage figures in the text should be standardized with the figures in the tables.

Line 315-316: The SD value is larger than the mean, indicating the probability of skewness.

Table 2 and 3: For the sample size n, indicate clearly which one represents 365, 366, 348, 349.

Random effects, ICC, model fit, etc., if any, are to be reported.

Line 303, Supplementary Tables S2: Given that multiple imputation was used, clarify how many imputed datasets were generated and whether all datasets were analyzed and pooled? This is to be clearly described.

Some references do not conform to the journal’s formatting requirements.

**********

6. PLOS authors have the option to publish the peer review history of their article (what does this mean?). If published, this will include your full peer review and any attached files.

If you choose “no”, your identity will remain anonymous but your review may still be made public.

**Do you want your identity to be public for this peer review?**  For information about this choice, including consent withdrawal, please see our Privacy Policy.

Reviewer #1: No

Reviewer #2: No

**********

[NOTE: If reviewer comments were submitted as an attachment file, they will be attached to this email and accessible via the submission site. Please log into your account, locate the manuscript record, and check for the action link "View Attachments". If this link does not appear, there are no attachment files.]

To ensure your figures meet our technical requirements, please review our figure guidelines: https://journals.plos.org/plosone/s/figures

You may also use PLOS’s free figure tool, NAAS, to help you prepare publication quality figures: https://journals.plos.org/plosone/s/figures#loc-tools-for-figure-preparation.

NAAS will assess whether your figures meet our technical requirements by comparing each figure against our figure specifications.

# Author comment: Author response to Decision Letter 1

**DOI:** 10.1371/journal.pone.0344453.r002

9 Jun 2026

Thank you for taking the time to review our manuscript. We are pleased to present this revised version, which incorporates your valuable feedback. In the following sections, we address each comment and provide clarification where needed. In the updated manuscript, we used the Track Changes tool to make all modifications easy to identify and reference.

Reviewer #1

Throughout

-Some inconsistencies with capitalization and abbreviations (white vs White, US vs. U.S.)

RESPONSE: We reviewed the manuscript and revised inconsistencies in capitalization and abbreviations to ensure adherence to journal style and consistency throughout the text (e.g., “White” and “U.S.”).

-Switch to person first language (people who smoke instead of smokers)

RESPONSE: We carefully reviewed the manuscript to use person-first language where appropriate, including replacing terms such as “smokers” with “people who smoke” throughout the document.

Abstract

-Can the authors describe what’s in the messages? Are they images or text based? What is the focus of the messages?

RESPONSE: We revised and adapted the abstract section to provide additional detail regarding each of the cigarette pack labeling conditions used in the study (Page 2, lines 23-27).

-Line 29 typo "...network smoking prevalence WAS positively associated..."

RESPONSE: We carefully reviewed the sentence and confirmed that the verb agrees with the compound subject (“network disapproval of smoking and network smoking prevalence were positively associated”). The sentence has been retained as written (Page 2, line 36).

-Did models adjust for study condition?

RESPONSE: We thank the reviewer for this important question. Yes, the adjusted models included the experimental message study condition as a control variable. We revised the Methods section in the Abstract to clarify that all adjusted models controlled for study condition and the other covariates included in the analyses (Page 2, lines 30 and 31).

Intro

Line 42- typo ‘influence affect behaviors’ take out ‘affect’

RESPONSE: We revised the sentence by removing the word “affect” for clarity and grammatical accuracy (Page 4, line 95).

Line 142-145- did you hypothesize that the association would be positive or negative?

RESPONSE: We thank the reviewer for this helpful suggestion. We revised the hypotheses section to explicitly state the expected direction of associations between network characteristics and conversations about smoking harms and quitting benefits. Specifically, we clarified the hypothesized negative associations for tie strength, smoking disapproval, and network smoking prevalence (Page 8, line 234).

Methods

-Were alters with ‘don’t know’ responses dropped from number or treated as non-smokers?

RESPONSE: We thank the reviewer for this important question. Alters with “Don’t know” responses were not counted as people who smoke in the network smoking variable. Specifically, the variable reflected the number of alters for whom participants responded “Yes” to the smoking status question. We revised the manuscript to clarify this point (Page 13, lines 367-368).

-Given that the study is from an experiment involving messaging, the authors should provide more detail about the specific messaging used.

RESPONSE: We revised the manuscript to provide additional detail regarding the experimental messaging conditions, including the format and focus of the efficacy-message inserts and pictorial health warning labels. Specifically, we clarified that participants in the insert conditions received small printed leaflets placed inside cigarette packs that included both text and imagery focused on the benefits of smoking cessation and strategies to support quitting. We also clarified that the PHWLs combined graphic images with text describing the harms of smoking and contained the same textual content as the text-only warning labels used in the control condition. We also note that the manuscript references the parent trial publication, which provides full details regarding the study protocol and messaging materials (Page 10, lines 275-281).

-Did authors check for multicollinearity (VIFs) between network size and number of smokers/disapprovers? Should be reported, if so.

RESPONSE: We assessed multicollinearity among all predictors, including network size, number of people who smoke within the network, and number of alters who disapprove of smoking, using the variance inflation factor (VIF). The results indicated no evidence of problematic multicollinearity. We have added this information to the Methods section (Page 15, lines 432-434).

Results

-Line 313 typo average NUMBER OF cigarettes

RESPONSE: We thank the reviewer for identifying this typo. We revised the text to correct the wording to “average number of cigarettes” (Page 17, line 509).

Discussion

Line 355- missing period at the end of paragraph

RESPONSE: We added the missing period at the end of the paragraph (Page 22, line 656).

Line 396- typo much LARGER samples

RESPONSE: We revised the text to correct the wording to “much larger samples” (Page 24, line 718).

Reviewer #2:

Line 150-154: The detailed inclusion and exclusion criteria are to be provided.

RESPONSE: We thank the reviewer for this suggestion. We revised the manuscript to provide a more detailed description of the inclusion and exclusion criteria. Specifically, we clarified participant eligibility requirements related to age, English language proficiency, smoking history and frequency, and smoking status verification, as well as exclusion criteria related to the use of other nicotine products (Page 9, lines 244-250).

Line 261: The coding of this variable is to be clearly stated.

RESPONSE: We thank the reviewer for this suggestion. We revised the manuscript to clarify the coding of the recent quit attempt variable. Specifically, responses were categorized as “Yes” or “No,” with “Don’t know” responses classified as missing (Page 14, lines 390-392).

Line 269: The symbol ; is to be replaced with a comma.

RESPONSE: In response to this and the next comment, we revised the text to simplify the presentation of the education categories and replaced semicolons with commas to improve readability (Page 14, line 400).

Line 270, Table 1: The order of education level is to be revised e.g. ≤ high school; technical school, some college, community college, or university degree or more.

RESPONSE: We revised the description of the education categories in the text and Table 1 to simplify the presentation and align them to improve clarity and consistency (Page 14, lines 400-401).

Line 277: Proper citation of the statistical software is to be given e.g. Stata 17 (StataCorp, College Station, TX, USA)

RESPONSE: We revised the manuscript to provide the proper citation for the statistical software used in the analyses: “Stata 17 (StataCorp, College Station, TX, USA)” (Page 15, line 413).

Line 280: The name of the statistical test is to be stated, e.g. the mixed effects logistic regression models were fitted using e.g. GLMM etc

RESPONSE: We thank the reviewer for this comment. We clarify that the analyses used generalized linear mixed models (GLMMs) with a logit link (i.e., mixed-effects logistic regression models) and random intercepts for participants to account for repeated EMA observations nested within individuals. We revised the model description and equation to use standard GLMM notation (Page 15, lines 415-417).

A statement on ‘Model assumptions for the statistical test, e.g., generalized linear mixed models, were satisfied is to be provided.

RESPONSE: We thank the reviewer for this suggestion. We revised the manuscript to include a statement regarding the evaluation of model assumptions and diagnostics for the generalized linear mixed models. Specifically, we added information indicating adequate model convergence, no evidence of problematic multicollinearity based on variance inflation factor (VIF) assessments, and no substantial departures from normality in the participant-level random intercepts, as indicated by visual inspection of Q-Q plots. We also clarified that intraclass correlation coefficients estimated separately for each outcome from the fully adjusted models indicated substantial clustering of repeated EMA observations within participants, supporting the inclusion of participant-level random intercepts. These additions have been incorporated into the Methods section (Pages 15 and 16, lines 432-476).

Line 286-288, 290-294: The paragraph is unclear and requires revision.

RESPONSE: The paragraph describing the mixed-effects logistic regression models was revised to improve clarity and consistency with standard generalized linear mixed model (GLMM) notation. Specifically, we clarified the multilevel structure of the data, including the nesting of repeated EMA observations within participants, the specification of participant-level random intercepts, the model equation using conventional GLMM notation, and the inclusion of both participant-level and observation-level covariates (Page 15, lines 423-432).

Line 289: The equation is commonly expressed in generalized linear mixed effects model (GLMM). A two-level mixed-effects logistic regression model with a random intercept is specified as logit(P(Yij=1))=β0+β1X1ij+⋯+βkXkij+u0j

RESPONSE: We thank the reviewer for this suggestion. We revised the model equation to align with the conventional specification of a generalized linear mixed model (GLMM) for a binary outcome. Specifically, the equation was reformulated using a logit link function and participant-level random intercepts to reflect the mixed-effects logistic regression models estimated in the analyses (Page 15, line 427). We also revised the accompanying text to improve clarity and consistency (Page 15, lines 423-432).

Line 298: The rationale of using multiple imputation is to be stated. Example the type of missingness is to be stated e.g. MAR, MCAR etc

RESPONSE: We appreciate this comment and have clarified the rationale for using multiple imputation. Because missingness in the dependent variables was associated with several observed covariates (i.e., network closeness, day in study, education, study period, age, and race), the missing-data mechanism was considered consistent with Missing At Random (MAR). We also clarified that multiple imputation was used to reduce potential bias associated with complete-case analyses and to investigate the effects of missingness on the study findings (Page 16, lines 480-483). The direction and significance of associations were the same in analyses using only observations with complete data and in analyses using imputed data; therefore, we report the results from the complete-case analyses.

Line 311 and other sections: The decimal point for the percentage figures in the text should be standardized with the figures in the tables.

RESPONSE: We revised the manuscript to standardize the presentation of percentage figures in the text, ensuring consistency with the formatting used in the tables. As a consequence, percentages were consistently reported to one decimal place throughout the manuscript (Pages 16 and 17, lines 495-509) and Table 1 (Pages 17 and 18).

Line 315-316: The SD value is larger than the mean, indicating the probability of skewness.

RESPONSE: We acknowledge that the standard deviations exceeded the corresponding means, indicating substantial variability and a likely right-skewed distribution of smoking- and cessation-related conversation frequency. The skewed distribution was acknowledged in the revised Results section (Page 17, lines 513-514). Because these outcomes reflected low-frequency repeated measures, such variability was expected in this sample.

Table 2 and 3: For the sample size n, indicate clearly which one represents 365, 366, 348, 349.

RESPONSE: We thank the reviewer for this suggestion. We revised Tables 2 and 3 (Pages 19 and 20) to clearly indicate the analytic sample sizes associated with each set of models. Specifically, we added table notes that identify the sample sizes for the bivariate, partially adjusted, and fully adjusted models and clarify minor differences across analyses due to missing data.

Random effects, ICC, model fit, etc., if any, are to be reported.

RESPONSE: We revised Tables 2 and 3 (Pages 19 and 20) to provide additional information regarding the mixed-effects models. Specifically, we noted that all models included participant-level random intercepts, reported the intraclass correlation coefficients (ICCs) estimated separately for each outcome from the fully adjusted models, and provided model fit statistics (AIC and BIC) for the fully adjusted models. Because Tables 2 and 3 examine different outcomes (conversations about smoking harms and conversations about quitting benefits, respectively), the corresponding ICC values differ across tables. We also clarified this information in the table footnotes.

Line 303, Supplementary Tables S2: Given that multiple imputation was used, clarify how many imputed datasets were generated and whether all datasets were analyzed and pooled? This is to be clearly described.

RESPONSE: We thank the reviewer for this suggestion. We revised the manuscript and Supplementary Table S2 (now Supplementary Table S1) to provide additional detail regarding the multiple imputation procedure. Specifically, we clarified that 20 imputed datasets were generated for each outcome using chained equations. We also clarified that all hypothesized relationships were re-estimated using the imputed datasets and that the resulting estimates were combined using standard multiple-imputation procedures (Page 16, lines 485–488 and Supplementary Tables S1).

Some references do not conform to the journal’s formatting requirements.

RESPONSE: We thank the reviewer for this observation. We carefully reviewed and revised the reference list to improve consistency and ensure conformity with the journal’s formatting requirements.

Attachment Submitted filename: Response to Reviewers.docx

# Editor report: Decision Letter 1

**DOI:** 10.1371/journal.pone.0344453.r003

**Authors:** David Zhu, David Zhu

23 Jun 2026

Associations of egocentric social network characteristics with conversations about the harms of smoking and benefits of quitting

PONE-D-26-04738R1

Dear Dr. Thrasher,

We’re pleased to inform you that your manuscript has been judged scientifically suitable for publication and will be formally accepted for publication once it meets all outstanding technical requirements.

Within one week, you’ll receive an e-mail detailing the required amendments. When these have been addressed, you’ll receive a formal acceptance letter and your manuscript will be scheduled for publication.

An invoice will be generated when your article is formally accepted. Please note, if your institution has a publishing partnership with PLOS and your article meets the relevant criteria, all or part of your publication costs will be covered. Please make sure your user information is up-to-date by logging into Editorial Manager at Editorial Manager® and clicking the ‘Update My Information' link at the top of the page. For questions related to billing, please contact billing support.

If your institution or institutions have a press office, please notify them about your upcoming paper to help maximize its impact. If they’ll be preparing press materials, please inform our press team as soon as possible -- no later than 48 hours after receiving the formal acceptance. Your manuscript will remain under strict press embargo until 2 pm Eastern Time on the date of publication. For more information, please contact onepress@plos.org.

Kind regards,

David T. Zhu

Academic Editor

PLOS One

Additional Editor Comments (optional):

The authors have addressed all comments brought forth by the reviewers. I am pleased to recommend acceptance.

Reviewers' comments:

# Editor report: Acceptance letter

**DOI:** 10.1371/journal.pone.0344453.r004

**Authors:** David Zhu, David Zhu

PONE-D-26-04738R1

PLOS One

Dear Dr. Thrasher,

I'm pleased to inform you that your manuscript has been deemed suitable for publication in PLOS One. Congratulations! Your manuscript is now being handed over to our production team.

At this stage, our production department will prepare your paper for publication. This includes ensuring the following:

* All references, tables, and figures are properly cited

* All relevant supporting information is included in the manuscript submission,

* There are no issues that prevent the paper from being properly typeset

You will receive further instructions from the production team, including instructions on how to review your proof when it is ready. Please keep in mind that we are working through a large volume of accepted articles, so please give us a few days to review your paper and let you know the next and final steps.

Lastly, if your institution or institutions have a press office, please let them know about your upcoming paper now to help maximize its impact. If they'll be preparing press materials, please inform our press team within the next 48 hours. Your manuscript will remain under strict press embargo until 2 pm Eastern Time on the date of publication. For more information, please contact onepress@plos.org.

You will receive an invoice from PLOS for your publication fee after your manuscript has reached the completed accept phase. If you receive an email requesting payment before acceptance or for any other service, this may be a phishing scheme. Learn how to identify phishing emails and protect your accounts at https://explore.plos.org/phishing.

If we can help with anything else, please email us at customercare@plos.org.

Thank you for submitting your work to PLOS ONE and supporting open access.

Kind regards,

PLOS ONE Editorial Office Staff

on behalf of

Mr. David T. Zhu

Academic Editor

PLOS One
